# Treatise on the Diseases of Women

**Published:** 1889-06

**Authors:** 


*Treatise on the Diseases of Women.* By ALEXANDER J. C. SKENE, M.D., of Brooklyn, New York. Pp. 966. London: H. K. LEWIS. 1889.

This is a large, ambitious, and important work by one well known as an authority on the subject it deals with. Type, illustrations, and paper are of the admirable excellence which so much add to the pleasure of perusing books brought out in America. The illustrations in particular are exceeding well done, and are mostly original.

Turning to the subject matter, the most striking feature is the independence of the writer. He speaks almost entirely from his own educated experience. Even if one differed from his teachings one is glad to welcome the honestly told narrative of a skilful and cultured worker in a field where much tilling has not always been followed by abundant fruit.

To traverse the whole range of the work would be impossible in a review extending over many pages. The most noteworthy chapters are those dealing with diseases of the bladder, in which the author has clearly been an original student to good purpose. Displacements of the uterus are sensibly and scientifically treated. In the various abdominal operations the author is specially a follower of Keith (to whom the work is dedicated), and he could have no better master. There are certain praiseworthy and judicious omissions, which in most modern works on women's diseases have a prominent position. To these omissions we would call special attention. They belong to the somewhat large class which have been begotten rather by a *pruritus secandi* than by a *spes medendi*.

On only one point would we venture to differ with some degree of confidence from our author; and that is on a point on which we believe most English surgeons would differ with our American brethren. There is too much assistance, human and instrumental, at all operations and examinations. For instance, we are told that for ovariotomy "three assistants are certainly needed, and one more may be required." What is the poor surgeon to do amongst so many? In the use of instruments Dr. Skene is far more sparing than most American gynæcologists: he recommends only two varieties of specula, and figures only four uterine sounds. Among the specula Fergusson's (not Ferguson's) is not mentioned; and we honour the author for the omission, although we think it one of the most generally useful of all.

We have real pleasure in recommending this work to our readers. It represents neither the premature work of the young gynæcologist seeking for practice nor the library production of the literateur seeking for reputation, but the simple, sagacious, outspoken opinions of one who has had much experience and made the most of it. This last one is by no means the least important of the noteworthy works on gynæcology which America has recently given to the world.

